# Geographic Distribution of Rabies Virus and Genomic Sequence Alignment of Wild and Vaccine Strains, Kenya

**DOI:** 10.3201/eid3008.230876

**Published:** 2024-08

**Authors:** Evalyne N. Wambugu, Gathii Kimita, Sarah N. Kituyi, Michael A. Washington, Clement Masakhwe, Lucy M. Mutunga, Gurdeep Jaswant, S.M. Thumbi, Brian C. Schaefer, John N. Waitumbi

**Affiliations:** Walter Reed Army Institute of Research–Africa, Kenya Medical Research Institute, Kisumu, Kenya (E.N. Wambugu, G. Kimita, C. Masakhwe, J.N. Waitumbi);; University of Embu, Embu, Kenya (E.N. Wambugu, S.N. Kituyi);; Dwight D. Eisenhower Army Medical Center, Augusta, Georgia, USA (M.A. Washington);; National Institutes of Health, Bethesda, Maryland, USA (M.A. Washington);; University of Nairobi, Nairobi, Kenya (L.M. Mutunga, G. Jaswant, S.M. Thumbi);; Washington State University, Pullman, Washington, USA (S.M. Thumbi);; Uniformed Services University, Bethesda (B.C. Schaefer)

**Keywords:** Rabies, rabies virus, lyssavirus, viruses, zoonoses, diversity, geographic isolation, antigenicity, whole-genome sequencing, Kenya, Africa

## Abstract

Rabies, a viral disease that causes lethal encephalitis, kills ≈59,000 persons worldwide annually, despite availability of effective countermeasures. Rabies is endemic in Kenya and is mainly transmitted to humans through bites from rabid domestic dogs. We analyzed 164 brain stems collected from rabid animals in western and eastern Kenya and evaluated the phylogenetic relationships of rabies virus (RABV) from the 2 regions. We also analyzed RABV genomes for potential amino acid changes in the vaccine antigenic sites of nucleoprotein and glycoprotein compared with RABV vaccine strains commonly used in Kenya. We found that RABV genomes from eastern Kenya overwhelmingly clustered with the Africa-1b subclade and RABV from western Kenya clustered with Africa-1a. We noted minimal amino acid variances between the wild and vaccine virus strains. These data confirm minimal viral migration between the 2 regions and that rabies endemicity is the result of limited vaccine coverage rather than limited efficacy.

Rabies, a viral disease that causes encephalitis, is consistently deadly in exposed humans who are not vaccinated or promptly treated with postexposure prophylaxis (PEP). Rabies was recognized in Egypt around 2300 BCE (*1*), and a written account of the disease was included in the Laws of Eshnunna in Mesopotamia (*2*). From that region, rabies spread to Europe and then to Africa, following patterns of human colonization (*3*). In 1768, rabies was described in the Americas, occurring as an epizootic in Boston (E.C. Ramsey, honors thesis, Macalester College, 2017, https://digitalcommons.macalester.edu/intlstudies_honors/28).

Rabies virus (RABV) is transmitted through the bite of an infected animal, which inoculates the virus at the bite site. The virus travels from the inoculation site to the central nervous system (CNS), where it multiplies and migrates to the salivary glands, after which another animal or human can be inoculated through a bite. RABV modifies the behavior of its host, which becomes exceptionally aggressive, thus increasing the odds of conflict and biting (*4*). Once clinical signs appear in an infected host, fatality is nearly always certain (*5*). The primary mechanism of lethality involves direct targeting of the CNS via mechanisms that remain poorly defined (*6*). 

RABV belongs to the genus *Lyssavirus*, which also includes Lagos bat virus, Mokola virus, Duvenhage virus, European bat virus 1 and 2, and Australian bat lyssavirus, among others (*7*). The primary viral reservoirs are members of the orders Carnivora (e.g., dogs) and Chiroptera (bats), whereas humans are dead-end hosts (*8*). Compared with other lyssaviruses, RABV is by far the most commonly reported (*9*), causing ≈59,000 human deaths annually (*10*), >99% of which are associated with dog-mediated transmissions (*9*). In rare human cases, rabies has been transmitted through non–bite-associated processes, such as organ transplants and laboratory exposures (*11*,*12*). 

Rabies has 2 major epidemiologic cycles: the urban cycle, in which dogs are the major reservoirs, and the sylvatic cycle, in which wild animals are the primary reservoirs (*13*). The urban cycle predominates in Africa, Asia, and Central and South America (*13*). The United States and some countries in Europe have used oral rabies vaccine programs to substantially reduce the sylvatic cycle (*14*). Nonetheless, rabies remains a global public health and veterinary challenge, particularly in developing countries (*15*).

RABV can be categorized into 2 major phylogenetic groups: bat-related and dog-related. Bat-related RABV is confined to the New World, but dog-related RABV is globally distributed (*16**–**18*). The dog-related group segregates into 6 distinct clades, which are designated as Africa-2, Asian, Africa-3, Arctic-related, Cosmopolitan, and Indian subcontinent (*19*). Three of those clades, Cosmopolitan (Africa-1 and Africa-4 subclades), Africa-2, and Africa-3, are found in Africa (*2*,*20*). The Cosmopolitan clade has >22 subclades that are widely distributed in >100 countries (*9*). The grouping of those clades is greatly affected by physical barriers that restrict gene flow (*21*). The Africa-1a subclade dominates in northern and eastern Africa, and Africa-1b predominates in central, eastern, and southern Africa. Africa-1c circulates in Madagascar, and Africa-4 was recently discovered in northern Africa (*22*). The Africa-2 clade predominantly circulates in West Africa, but the Africa-3 clade is sustained through a sylvatic cycle in South Africa (*19*,*22*).

Kenya has a >100-year history of rabies; the first case was reported in 1912 (*23*). Despite successful control efforts in the past, the disease has remained endemic because of challenges in sustaining vaccination programs (*24*,*25*). The number of human deaths attributable to rabies in Kenya is unknown (*23*), but some studies report estimates of >2,000 per year and others as low as 523 (*26*,*27*); the highest incidences have been reported in western and eastern Kenya (*23*). The main source of human rabies is the domestic dog (*28*). However, those human deaths are unjustifiable considering the availability of effective mass dog vaccination and therapeutic PEP for persons bitten by rabid animals (*25*,*29*). Kenya has an ambitious plan to end human rabies deaths by 2030; that plan combines mass dog vaccination and prompt provision of PEP to affected humans (*26*). Unfortunately, only 5% of health facilities have PEP, and mass dog vaccination has fallen short of the target (*25*,*26*). 

RABV, a negative-stranded RNA virus with a genome size of ≈12 kilobases, encodes 5 proteins: nucleoprotein (N), matrix protein (M), phosphoprotein (P), glycoprotein (G), and large (L) protein or polymerase (*30*,*31*). The N, P, and L proteins comprise the ribonucleoprotein (RNP) complex and the M and G proteins are involved in virus assembly and budding (*32*). The N gene is responsible for transcription and replication and shows antigenic variation that is used for strain discrimination (*7*,*33*,*34*–*36*). Since the 1880s, when Louis Pasteur developed a RABV vaccine (*37*), vaccination has played a crucial role in controlling rabies through production of neutralizing antibodies against the RNP and G antigens (*38*,*39*). G and N are crucial components for rabies vaccine effectiveness (*38*,*39*), and monitoring antigenic variations between vaccine strains and the G and N genes of circulating wild strains can help determine whether the existing vaccine strains remain effective (*33*,*40*). We assessed genetic diversity of wild RABV strains collected from 2 rabies hotspots in Kenya to inform current and future vaccination and PEP efforts.

## Methods

### Study Areas and Sample Cohort 

**Figure 1:**
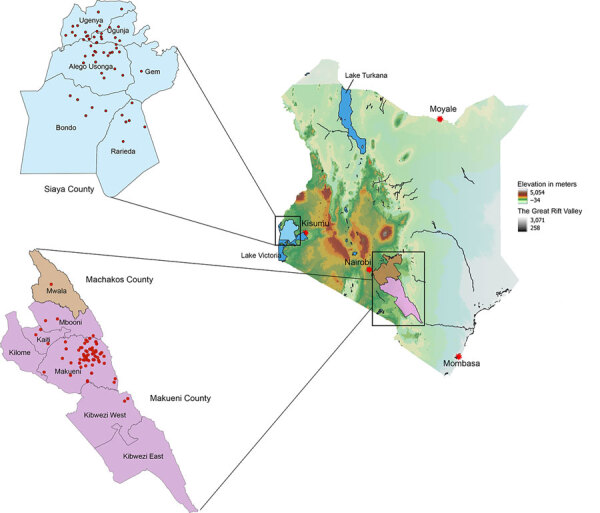
Geographic distribution of rabies virus from a study of genomic sequence alignment of wild and vaccine strains, Kenya. Red dots on callout maps indicate study sample collection sites in Siaya County in western and Makueni County in eastern Kenya. The 2 counties are 540 miles apart and separated by complex natural barriers, such as the floor of the Great Rift Valley, 6 lakes along the valley’s floor, the Aberdares and Nandi escarpment to the east, and the Lake Victoria to the west. Red asterisks indicate major cities or towns.

Veterinary technicians collected postmortem brain stems from suspected rabid animals in Kenya’s Siaya County, in the western region, and Makueni County, in the eastern region (Figure 1). Sample collection was approved by the Kenya Medical Research Institute’s Scientific and Ethical Research Unit under a rabies surveillance protocol (no. KEMRI/SERU/CGHR 046/3268).

We used Anigen Rapid Rabies Ag Kits (BioNote Inc., https://www.bionote.co.kr) to confirm RABV at the collection sites. That assay has a sensitivity of 92% (95% CI 85.9%–95.6%) and specificity of 100% (95% CI 93.4%–100%) compared with the reference standard fluorescent antibody test and is recommended for confirming clinical rabies cases in animals (*41*). We collected a total of 164 RABV-positive brain stems. We transported brainstems to the laboratory in liquid nitrogen and stored at −80°C until we performed nucleic acid purification.

### Nucleic Acid Isolation

We used the Bullet Blender (Next Advance, https://www.nextadvance.com) to homogenize ≈20 mg brainstem with 3-mm ceramic beads in TRIzol reagent (Thermo Fisher Scientific, https://www.thermofisher.com). We cooled samples at room temperature before centrifuging at 12,000 × *g* for 3 minutes at 4°C. We used 200 μL of the aqueous phase to extract nucleic acids by using MagMAX CORE Purification Kit (ThermoFisher Scientific), then eluted extracted nucleic acids in a total volume of 60 µL.

### Molecular Detection of RABV by qRT-PCR

We used quantitative reverse transcription PCR (qRT-PCR) to screen nucleic acids for RABV by targeting the large (L) gene using forward primer 5′-GGTTTCCGGDGCYGTDCCTC-3′, reverse primer 5′-CCTAGGGGAGACYTTGCCRT-3′ primer, and a 6FAM-CCCGTCAYATAGGGTCRGCTCARGGGC‐BBQ probe. The qRT-PCR reaction comprised 4 μL of the nucleic acid, 10 μL of 2x SensiFAST Master mix (Meridian Bioscience, https://www.meridianbioscience.com), 0.8 μL forward and reverse primer mix at a concentration of 10 μM each, 0.4 μL of TaqMan QSY probe (Applied Biosystems/Thermo Fisher Scientific) at a concentration of 10 μM, 3.4 μL of nuclease-free water, 0.4 μL RiboSafe RNase inhibitor (Thermo Fisher Scientific), and 0.2 μL reverse transcriptase. We used an ABI 7500 (Applied Biosystems) for amplification using a reverse transcription cycle at 45°C for 10 minutes and 95°C for 3 minutes to inactivate the reverse transcriptase, followed by 40 cycles of 95°C for 30 seconds and 60°C for 1 minute. Each reaction included a positive control of a known RABV-positive sample and PCR water as a nontemplate negative control.

### Whole-Genome Sequencing

We used the TURBO DNase kit (ThermoFisher Scientific) to deplete extracted nucleic acids of host genomic DNA. We used sequence-independent, single-primer amplification to amplify viral RNA as previously described (*42*), with subsequent modifications (*43*). We then reversed transcribed the first cDNA strand by using the LunaScript RT SuperMix kit (New England Biolabs, https://www.neb.com) and the JH17N8 primer (5′-GTTTCCCAGTAGGTCTCNNNNNNNN-3′), which contained a degenerate 8-mer sequence at the 3′ end. We generated the second cDNA strand by amplification using NEB Next Ultra II Q5 master mix (New England Biolabs) with 10 μM of a specific primer JHP21 (5′-GTTTCCAGTAGGTCTC-3′) and the following thermal cycling regimen: 1 cycle of 94°C for 3 minutes, 25°C for 30 seconds, 72°C for 1 minute, followed by 35 cycles of 94°C for 30 seconds, 55°C for 30 seconds, and 72°C for 1 minute. We included a final extension step of 72°C for 1 minute, followed by cooling to 4°C. We purified the synthesized dsDNA by using Agencourt AMPure XP beads (Beckman Coulter, https://www.beckmancoulter.com), which we then used to prepare sequence libraries using the Colibri ES DNA Library preparation kit (ThermoFisher Scientific). We quantified libraries by using a Qubit dsDNA HS Assay kit (Invitrogen/Thermo Fisher Scientific) and measured the average library size on the 4200 TapeStation System (Agilent Technologies, https://www.agilent.com). We adjusted the pooled library to 4 nM concentration and then denatured with 0.2 normal sodium hydroxide and further diluted to 9.5 pM. We spiked 5% of PhiX v3 Control Library (Illumina, https://www.illumina.com) into the pool and sequenced the library in pairs using 600 cycles v3 on the Miseq or P3 reagents on the NextSeq 2000 platform (Illumina).

### Genome Assembly, Clade Classification, and Phylogenetic Reconstruction

We assessed the quality of the raw sequences in FastQC v0.12.1 (https://github.com/s-andrews/FastQC/releases) and then processed by using the ngs_mapper pipeline (https://github.com/VDBWRAIR/ngs_mapper), which removes low-quality reads (<Q20), failed reads, sequencing adapters, and short reads (<50 nt) by using trimmomatic v0.35 (https://github.com/usadellab/Trimmomatic/releases) and cutadapt v1.9.1 (https://gensoft.pasteur.fr/docs/cutadapt/1.9.1/index.html). We then mapped the filtered reads against a RABV genome from Tanzania (GenBank accession no. KY210291) that had the closest homology to our sample sequences by using bwa v0.7.12 (https://github.com/lh3/bwa/releases). We used Samtools v0.1.19 (https://sourceforge.net/projects/samtools/files/samtools/0.1.19) to create pileups from the read alignments and the consensus genome and generated a variant call format file and coverage visualizations by using several Python scripts (Python Software Foundation, https://www.python.org) housed within the pipeline.

To determine whether the generated whole genomes add value to genomes generated from the N and G genes, we used the RABV-GLUE tool (http://rabv-glue.cvr.gla.ac.uk) to assign the RABV to major and minor clades. Further classification of the whole genomes into lineages was performed using MADDOG (*44*).

To establish the phylogenetic relationships between RABV from Kenya in the context of Africa, we obtained a comprehensive subset of curated, annotated, and published RABV datasets from Africa from the Bacterial and Viral Bioinformatics Resource Center (BV-BRC; https://www.bv-brc.org). We aligned the complete RABV polyprotein, the entire N protein, and the entire G protein of the study genomes and context samples in CLC Genomics workbench version 8.5.1 and the Muscle plugin (QIAGEN, https://www.qiagen.com). To avoid using sequences from recombinations, we ran the aligned sequences in GARD software (Datamonkey, https://www.datamonkey.org) by using the Hyphy package (Datamonkey). We performed phylogenetic inference by using the maximum-likelihood method in IQ-TREE version 2.2.0 (http://www.iqtree.org) and nucleotide substitution models built into ModelFinder in IQ-TREE. We evaluated node support with a combination of approximate likelihood tests and ultrafast bootstraps with 1,000 replicates, each computed in IQ-TREE (*45*). We visualized and annotated the resulting phylogenetic trees by using FigTree version 1.4.2 (http://tree.bio.ed.ac.uk/software/Figtree).

### Analysis of Amino Acid Variation at RABV Vaccine Target Sites

To identify amino acid variations at the N and G vaccine target sites of the study genomes and the vaccine strains, we performed sequence alignment of the G (n = 142) and N (n = 144) proteins by using CLC Genomics Main Workbench (QIAGEN). We aligned study sample sequences to 3 commonly used RABV vaccine strains: Pitman-Moore (PM; GenBank accession no. DQ099525), Pasteur virus (PV; GenBank accession no. M13215), and challenge virus standard (CVS; accession no. AF406696 for N and AF406694 for G).

## Results

### Study Sample Demographics

Domestic dogs contributed most (65%) study samples, followed by cows (18%), and goats (14%). Other species accounted for only 1% of samples (Appendix 1 Table 1)

### RABV Geographic Restriction in Kenya

**Figure 2:**
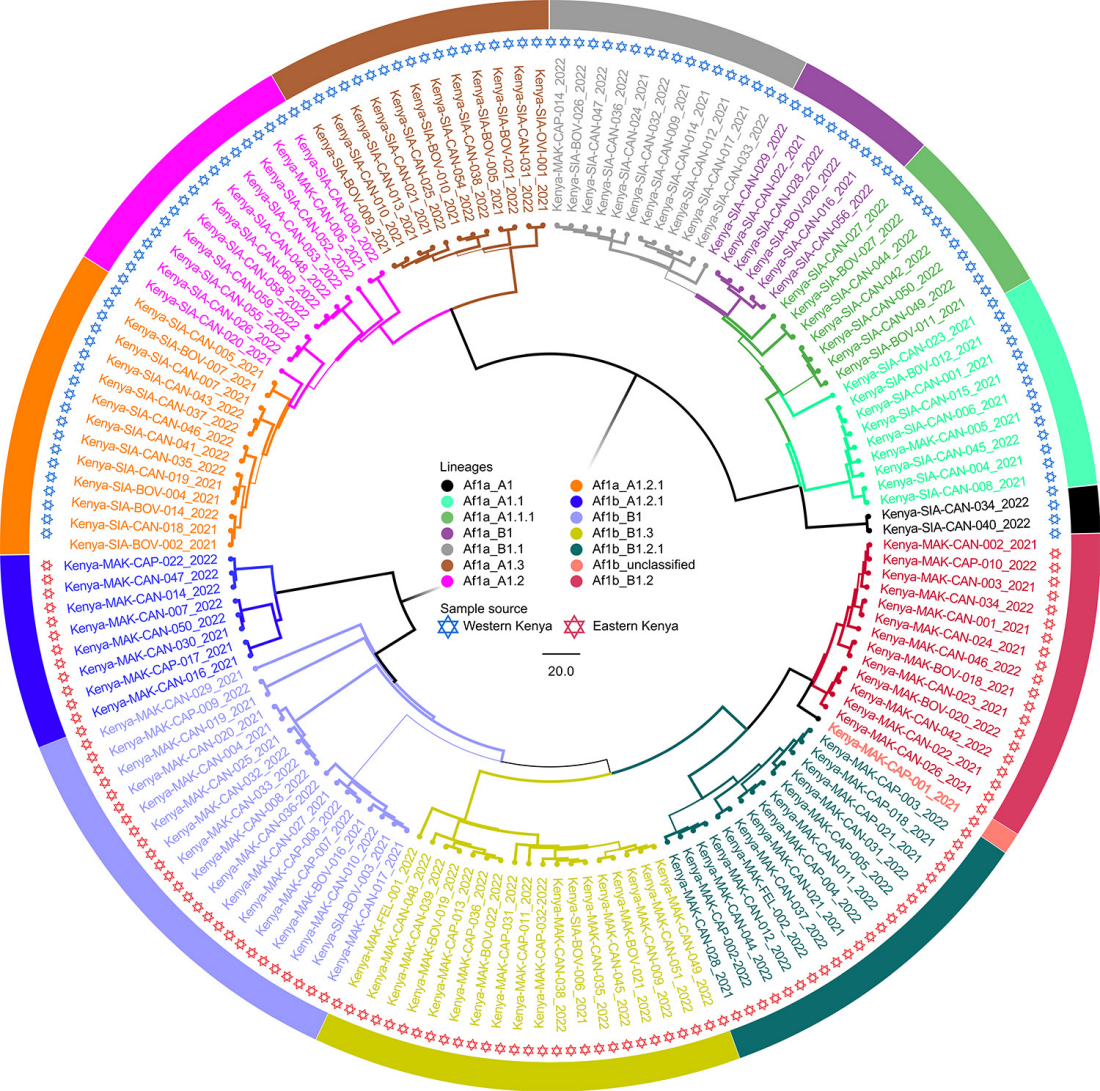
Phylogenetic tree of virus genomes from a study of geographic distribution of rabies virus and genomic sequence alignment of wild and vaccine strains, Kenya. The tree was constructed from whole-genome sequences by using MAD DOG (*44*). All genomes were of Cosmopolitan clade, subclades Africa-1a or Africa-1b, and were further delineated into a total of 14 distinct lineages, 8 from western Kenya and 5 in eastern Kenya. One lineage from eastern Kenya was unclassified. Scale bar indicates nucleotide substitutions per site.

Of the 164 brainstems collected, 144 samples with genome lengths ranging from 10,024 to 11,923 nt were used for whole-genome analysis. From whole-genome sequences, we also extracted 142 N and 144 G genes for single-gene analysis. By RABV-GLUE, all genomes belonged to the Cosmopolitan clade, either Africa-1a (71/144) or Africa-1b (73/144) subclades. Further interrogation of the clades using the MAADOG lineage typing tool revealed 14 distinct lineages, 8 in western Kenya, 6 in eastern Kenya, including 1 unclassified lineage in eastern Kenya (Figure 2).

We accessed the BV-BRC database on November 18, 2022, and retrieved 378 RABV polyproteins, 1,500 N, and 130 G sequences that met our inclusion criteria. We used RABV sequences from the study strains and strains subsampled from Africa to construct whole-genome phylogenetic trees (Appendix 2 Figures 1–3) and individual G and N gene trees (Appendix 2 Figures 4, 5). We compared the phylogenetic trees constructed using a relatively new method of whole-genome sequencing (has only few sequences in literature) with those generated from the old method of individual G and N genes analysis that has been the basis of RABV phylogeography. Both methods identified only the Cosmopolitan clade in our study genomes (Appendix 2 Figures 2, 4, 5). In addition, western Kenya samples (97.2%) branched with the Cosmopolitan Africa-1a subclade, and eastern Kenya samples (95.7%) branched with Cosmopolitan Africa-1b subclade (Appendix 2 Figures 2, 4, 5). Only 2 western Kenya genomes clustered with the Africa-1b subclade (Appendix 2 Figure 1) and only 3 eastern Kenya genomes clustered with the Africa-1a subclade (Appendix 2 Figure 3).

Within the Africa-1b subclade, the eastern Kenya samples appeared to have 2 distinct clusters, 1 major (n = 63) and 1 minor (n = 8) (Appendix 2 Figure 1). In both clusters, the study samples branched with Tanzania genomes. The western Kenya genomes (n = 69) were more homogeneous (Appendix 2 Figure 3). We deposited raw sequence data from this study in the National Center for Biotechnology Information (NCBI; https://www.ncbi.nlm.nih.gov) Sequence Read Archive (BioProject nos. OR256801 and OR270967–1061). 

### Comparison of Circulating Wild RABV and Vaccine Strains

Compared with sequences of the commonly used RABV vaccine strains in Kenya (PM, PV, and CVS), we noted no amino acid substitutions at the antigenic sites of the G gene, located between amino acids 20–439 (Appendix 1 Table 2). However, sequence homology outside the antigenic sites varied from 92.2% for PM to 93.3% for CVS and 93.0% for PV. In contrast, 2 study samples, Kenya/SIA-CAN-018/2021 and Kenya/SIA-BOV-007/202, were variant at antigenic site II of the N gene, in which alanine was substituted by valine at position 315 (A315V), but the 3 vaccine strains and all the other study samples had alanine in that position. In addition, although all the study samples, PM, and CVS had valine at position 379 of antigenic site III, PV was variant with alanine (V379A). Outside the antigenic sites of the N gene, sequence homology ranged from 98.45% to 99.11% for PM, 97.78% to 98.67% for CVS, and 97.56% to 98.23% for PV.

## Discussion

In this study, we used samples collected from 2 geographically isolated counties, Siaya in western Kenya and Makueni in eastern Kenya. We chose those 2 counties because they continuously report the highest rabies incidences in humans (*23*). Of the 164 brainstem study samples, 107 were from domestic dogs (Appendix 1 Table 1). Moreover, the other 57 samples came from animals with a history of having been bitten by a rabid dog, underscoring the role domestic dogs play in rabies transmission.

Previous phylogenetic studies, performed using single G and N genes, have indicated that RABV in Africa falls into several regional groups and that viruses from eastern Africa are genetically distinct from those in the western, central, and southern parts of the continent (*21*). Before this study, NCBI included 43 RABV sequences from Kenya (*46**–**48*). In a previous study (*46*), N and G genes from RABV samples obtained during an outbreak of rabies in African wild dogs in Kenya and Tanzania appeared to be identical. The authors concluded that the outbreak was most likely caused by a viral variant frequently found in domestic dogs in Kenya and Tanzania. A subsequent study that used N and G gene sequences reported existence of 2 Cosmopolitan subclades (*47*), namely Africa-1a and Africa-1b. Subsequent research revealed predominance of Africa-1a in western Kenya, but Africa-1b was more commonly observed in the eastern part of the country (*48*).

We added 144 whole-genome sequences from this study to the NCBI Sequence Read Archive. We used those genomes to further characterize the diversity of RABV from eastern and western Kenya. We also determined whether available rabies vaccines would confer protection. Our study confirms the inferences of previous research that shows an apparent geographic isolation between the RABV strains in eastern and western Kenya (*48*). Of the 3 major clades of RABV found in Africa, only the Cosmopolitan clade was detected by whole genomes (Appendix 2 Figures 1–3) and individual N and G genes (Appendix 2 Figures 4, 5). Predominantly, RABV from eastern Kenya clustered with the Africa-1b subclade, whereas RABV from western Kenya clustered with the Africa-1a subclade. That geographic isolation is probably because of the multiple landscape features that would restrict free movement of animals between the regions (Figure 1), thus promoting localized viral evolution. Similar geographic restriction has also been observed in raccoon-mediated rabies in the eastern United States (*49*). Outlier Africa-1b genomes that were in western Kenya (n = 2), and Africa-1a genomes (n = 3) that were in eastern Kenya indicate that geographic isolation is not absolute in the country (Appendix 2 Figures 1–3).

We found that RABV strains in western Kenya were closely related to each other and dissimilar from other Africa-1a subclade members in neighboring countries (Appendix 2 Figures 1–3). For example, the closest genome that was clearly distinct from the Kenya 1a subclade was from Sudan. Those observations suggest that the members of the Africa-1a circulating in Kenya have evolved separately, most likely from northern, central, and western regions of Africa. The other likely explanation is undersampling given that our study samples were collected in 2021 and 2022, but the other Africa-1a subgenomes were collected during 1986–2015. The Africa-1b genomes from Kenya were less homogeneous and branched into 2 groups: a major group of 63 genomes that clustered together and a minor group of 8 genomes that clustered with genomes that had been collected previously from Kericho and Nakuru, Kenya. However, using the MADDOG lineage typing tool, the western Kenya 1a subclade that was apparently homogeneous revealed 8 lineages, and the eastern Kenya subclade 1b segregated into 5 lineages (Figure 2), indicating that RABV accumulates mutations through the course of transmission. The finding of Africa-1b in Nakuru and Kericho, locales that are close to western Kenya, indicates an ongoing encroachment of the 1b subclade into western Kenya.

Several strains of RABV are used to manufacture vaccines (*38*). The choice of vaccine strain for use in a geographic region is informed by factors such as the circulating wild variants and species of animals being vaccinated (*50*). Live attenuated vaccines are given orally and are used in wild carnivores. Inactivated vaccines are used in humans and domestic animals, including dogs and cats. From the RABV genomes in our study, we evaluated whether the currently used RABV vaccines would confer protection (Appendix 1 Tables 2, 3). In Kenya, the available vaccines are derived from the PM, CVS, and PV RABV strains. An amino acid sequence alignment and comparison of the G sequences of the study genomes and the vaccine strains revealed 100% homology to the vaccine antigenic sites (Appendix 1 Table 2). A similar alignment and comparison using the N gene revealed a V315A replacement at antigenic site II in only 2 study samples. In addition, all the study samples and PM and CVS strains had valine at position 379 of antigenic site III, but the PV was variant with V379A (Appendix 1 Table 3). Although those replacements are too infrequent to effect vaccine efficacy, they raise concerns of potential cumulative changes that could eventually alter vaccine efficacy and underscore the need for continued monitoring of such changes.

In conclusion, we used whole-genome sequencing to define the genetic diversity of RABV in Kenya. Our data demonstrated the presence of localized viral lineages and limited viral migration between the 2 study regions. In addition, obtained data suggest that rabies endemicity is due to limited vaccine use because the sequences of the study strains do not greatly diverge from current vaccine strains. Moving forward, similar studies should expand to the other regions of Kenya to determine the generalizability of our findings. Nonetheless, the viral migration across the regions, though limited, reinforces the need for cross-county rabies surveillance systems in Kenya. 

Appendix 1 Genomic information on wild rabies virus strains and genomic sequence alignment with vaccine strains, Kenya.

Appendix 2 Additional information on geographic distribution of rabies virus and genomic sequence alignment of wild and vaccine strains, Kenya.
