# Supplementary material for: Geographic Distribution of Rabies Virus and Genomic Sequence Alignment of Wild and Vaccine Strains, Kenya
Source: Emerg Infect Dis. 2024 Aug;30(8):1642–50. doi: 10.3201/eid3008.230876 (PMC11286075; doi:10.3201/eid3008.230876)
Supplement: Appendix 2 — Additional information on geographic distribution of rabies virus and genomic sequence alignment of wild and vaccine strains, Kenya. [file 23-0876-Techapp-s2.pdf]

Article DOI: https://doi.org/10.3201/eid3008.230876

EID cannot ensure accessibility for supplementary materials supplied by authors. Readers who have difficulty accessing supplementary content should contact the authors for assistance.

Geographic Distribution of Rabies Virus and Genomic Sequence Alignment of Wild and Vaccine Strains, Kenya

Appendix 2

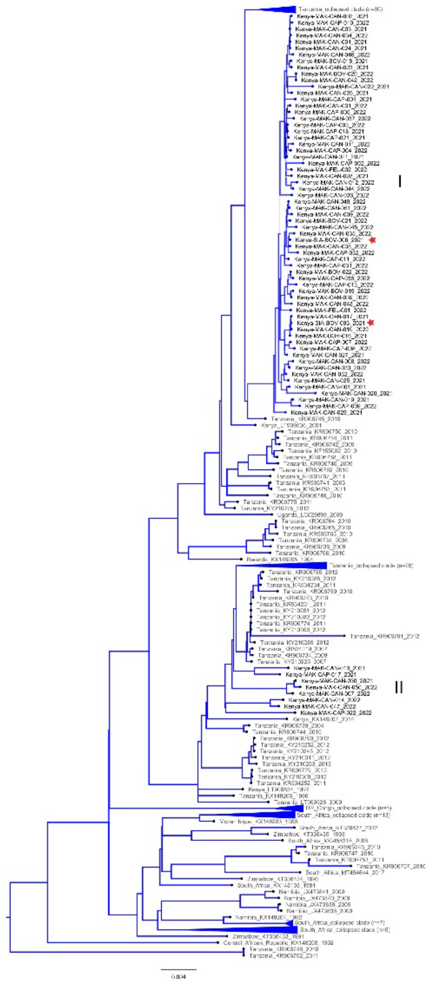

Appendix 2 Figure 1. Whole genome phylogenetic tree showing phylogeographic clade and subclade classification of study genomes compared to other African genomes. Two western Kenya genomes, shown in red stars clustered with the Africa-1b. Scale bar indicates nucleotide substitutions per site.

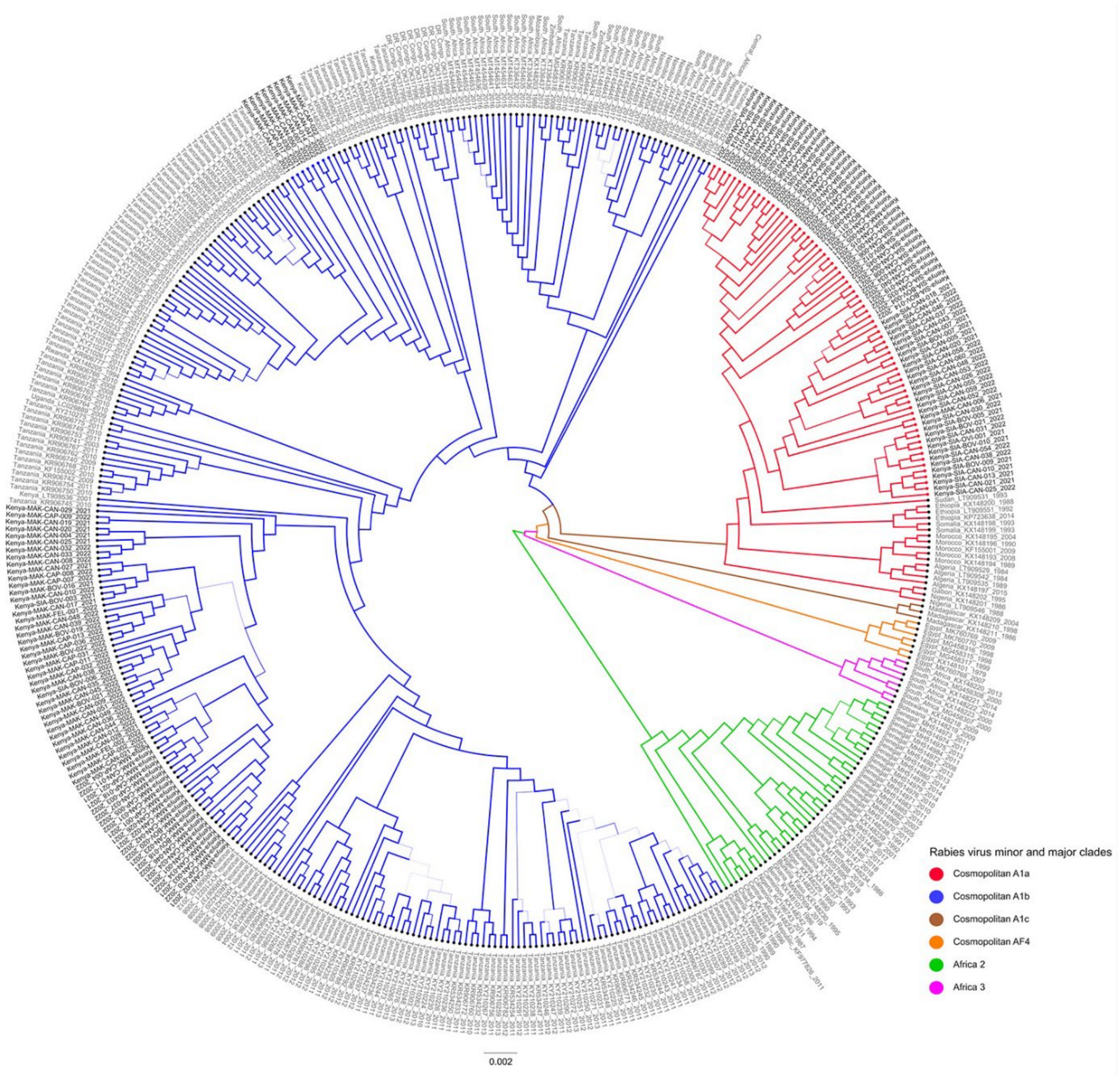

Appendix 2 Figure 2. Whole genome phylogenetic tree showing phylogeographic clade and sub-clade classification of study genomes compared to other African genomes. All Kenyan genomes were of Cosmopolitan clade (Appendix 2 Figure 2), subclades Africa-1a (western Kenya, shown as red lines, with the prefix “Kenya-SIA”) or Africa-1b (eastern Kenya, shown as blue lines, with the prefix “Kenya-MAK”). Scale bar indicates nucleotide substitutions per site.

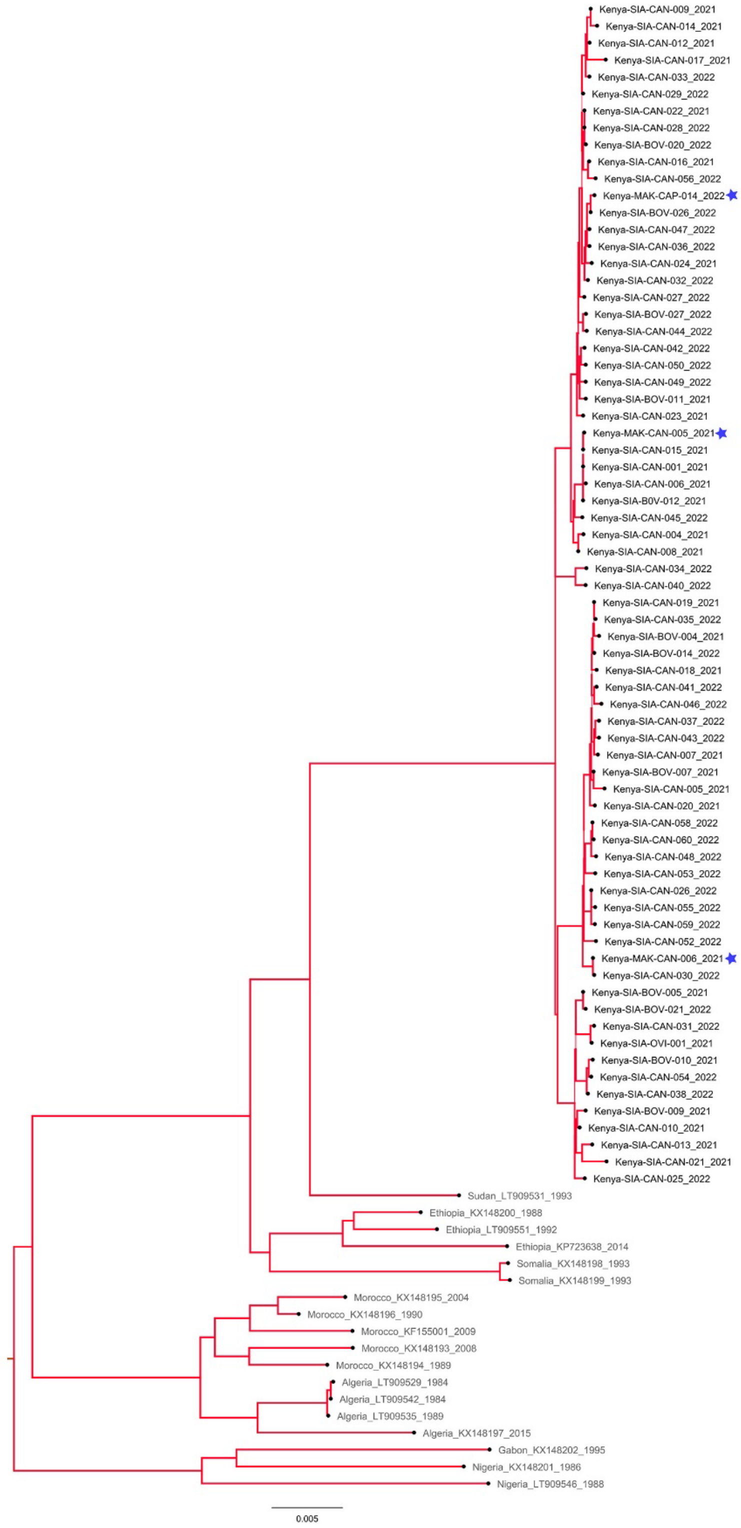

Appendix 2 Figure 3. Whole genome phylogenetic tree showing phylogeographic clade and sub-clade classification of study genomes compared to other African genomes. Three eastern Kenya genomes, shown in blue stars clustered with the Africa-1a. Scale bar indicates nucleotide substitutions per site.

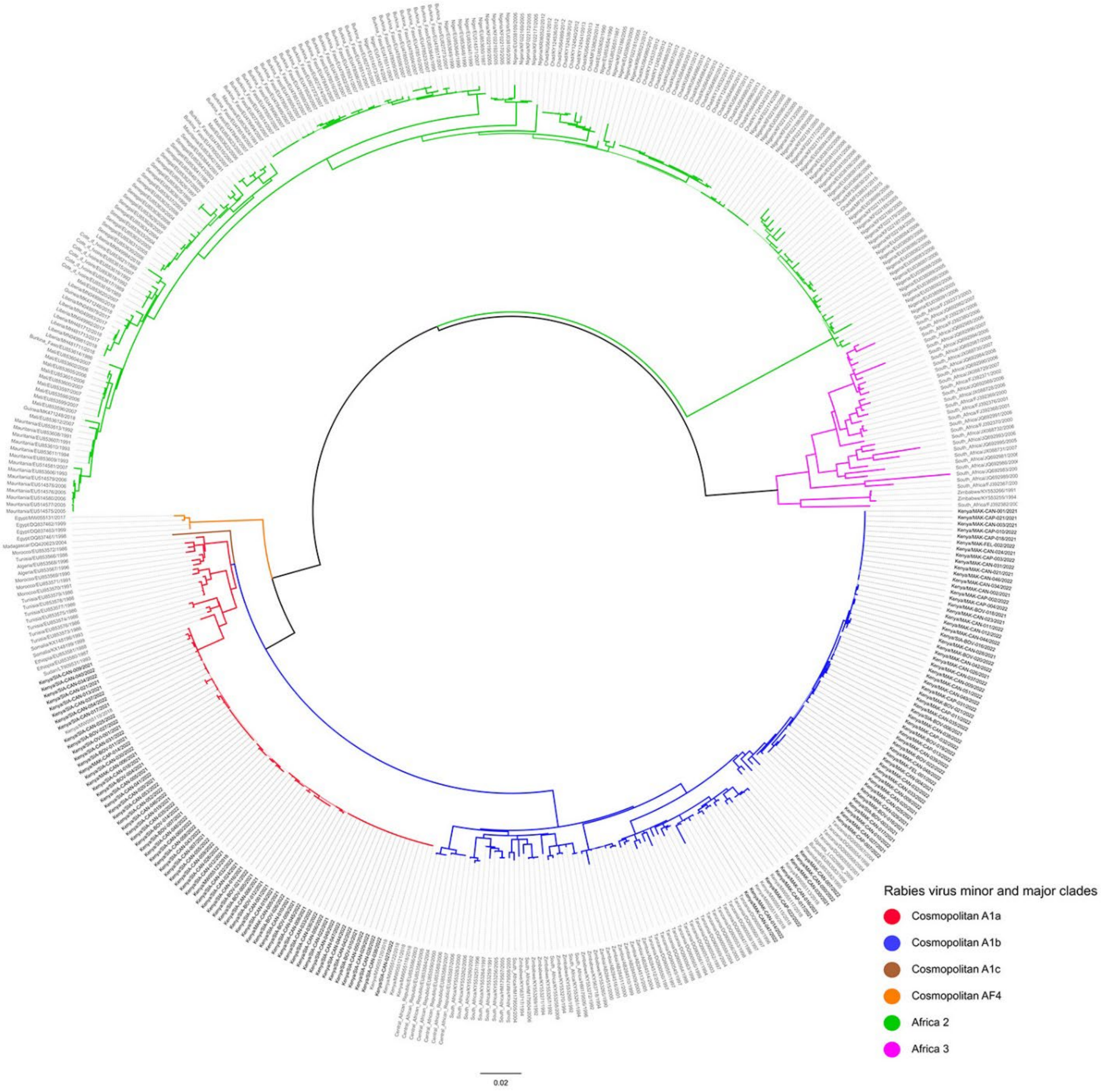

Appendix 2 Figure 4. Phylogenetic trees constructed with individual N genes. Unlike the tree drawn from whole genome, the single gene trees only identified the virus to subclade level: Africa-1a (shown in red lines) and sub-clade Africa-1b (shown in blue lines) but failed to identify lineages. Scale bar indicates nucleotide substitutions per site.

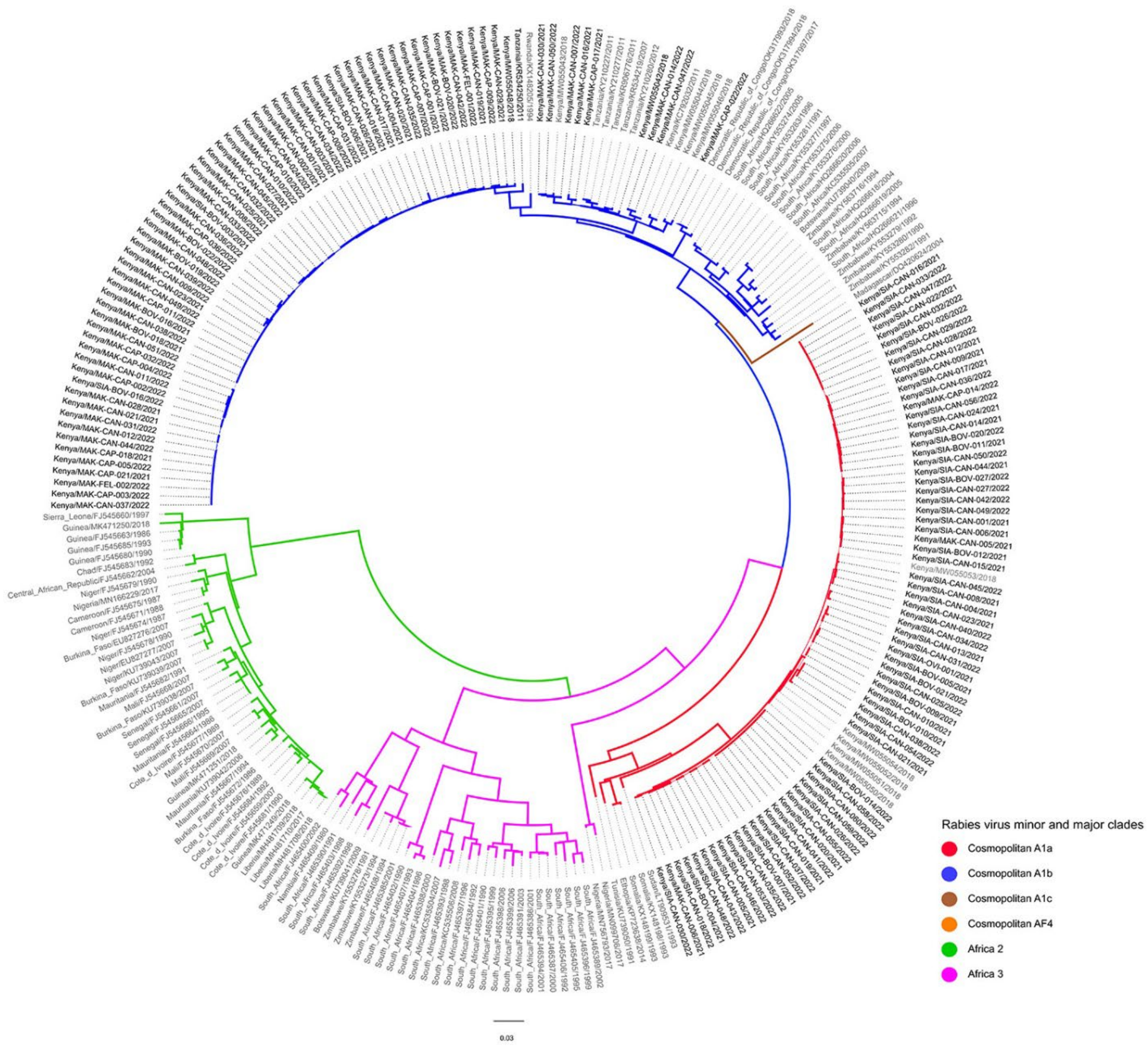

Appendix 2 Figure 5. Phylogenetic trees constructed with individual G genes. Unlike the tree drawn from whole genome, the single gene trees only identified the virus to subclade level: Africa-1a (shown in red lines) and sub-clade Africa-1b (shown in blue lines) but failed to identify lineages. Scale bar indicates nucleotide substitutions per site.
